# Temperature-induced hemoglobin dynamics: oxygenation-dependent evolution of hemoglobin spectral response in the near-infrared

**DOI:** 10.1117/1.BIOS.3.2.025004

**Published:** 2026-05-25

**Authors:** Alessandro Bossi, Fabio Negretti, Paola Saccomandi, Antonio Pifferi

**Affiliations:** a Politecnico di Milano, Department of Physics, Milan, Italy; b Politecnico di Milano, Department of Mechanical Engineering, Milan, Italy

**Keywords:** diffuse optics, time-domain, thermal treatment, hemoglobin, absorption coefficient

## Abstract

**Significance:**

Monitoring thermally induced optical changes can guide thermal therapies, but these signals depend strongly on hemoglobin oxygenation, leading to different spectral responses under *ex vivo* and *in vivo* conditions. Clarifying these effects is essential for interpreting absorption measurements during treatment.

**Aim:**

We characterized near-infrared absorption spectra of hemoglobin during heating under controlled oxygenated and deoxygenated conditions and related the signatures to hemoglobin chemistry.

**Approach:**

Using time-domain diffuse optical spectroscopy, we measured 680- to 1110-nm absorption spectra of blood phantoms containing oxyhemoglobin (oxyHb) or deoxyhemoglobin (deoxyHb) during controlled heating. Multi-concentration measurements yielded extinction coefficients for native and thermally denatured species.

**Results:**

Heating oxyHb caused a broad absorption rise consistent with autoxidation of ferrous (${\mathrm{Fe}}^{2+}$) heme to ferric (${\mathrm{Fe}}^{3+}$) forms, similar to methemoglobin and hemichromes. Heated deoxyHb showed distinct peaks at 774 and 874 nm that disappeared upon re-oxygenation, after which spectra converged to treated oxyHb. The oxygen-dependent kinetics of the 774/874 nm bands support a ferrous-denatured intermediate that converts to ferric hemichromes when oxygen becomes available.

**Conclusions:**

Thermal denaturation of hemoglobin follows distinct oxygenation-dependent spectroscopic pathways. The extracted extinction coefficients provide essential inputs for optical models of laser-based ablation and improve diffuse optical monitoring of thermal therapies.

Statement of Discovery Using time-domain diffuse optical spectroscopy, this study reveals oxygenation-dependent spectral pathways of hemoglobin during thermal treatment. Thermally treated deoxyhemoglobin exhibits two transient absorption peaks at 774 and 874 nm that disappear upon re-oxygenation, after which the spectra converge to those of treated oxyhemoglobin. These observations suggest the formation of a ferrous-denatured intermediate characterized by the 774/874-nm bands that, upon oxygen availability, converts to ferric hemichromes, informing optical monitoring and laser-based thermal therapies.

## 1 Introduction

Hemoglobin (Hb) is the primary oxygen transport molecule in the human body and is also responsible for the characteristic red color of blood. Its optical properties are highly subject to changes following exposure to heat. In this work, we investigate how the absorption spectra of Hb evolve under different oxygenation states during heating.

Our final goal is to develop, through optical methods, an effective tool to monitor thermal therapies, such as laser ablation,^1^ microwave ablation,^2^ or radiofrequency ablation,^3^ which are minimally invasive methods^4^ with great potential for treating a variety of pathologies, including localized tumors^5^^–^^8^ or atrial fibrillation.^9^ Compared with major surgery, they can significantly improve patient quality of life as they reduce pain, have fewer complications, and allow for a faster recovery.^10^ The lack of an effective tool to monitor these therapies and to control the ablation zone is a critical limitation for real-time treatment assessment.^11^ Although existing approaches, such as ultrasound^12^ and thermometry,^12^^–^^17^ offer some benefits for monitoring thermal therapies, they face critical limitations. These challenges hinder their ability to precisely classify the extent of thermal damage.

Optical methods, whose signatures directly relate to tissue damage, have gained attention. Techniques such as Raman spectroscopy,^18^ hyperspectral imaging,^19^^,^^20^ and optical coherence tomography^21^ offer high specificity while remaining non-invasive but are constrained by the limited depth penetration (<1  mm). In contrast, diffuse optical approaches, which estimate the absorption coefficient ($\mu_{a}$) and reduced scattering coefficient ($\mu_{s}^{\prime }$) offer greater depth sensitivity while remaining non-invasive. Therefore, these techniques hold significant potential for use in monitoring and modeling thermal treatments. As diffuse optics is inherently sensitive to hemoglobin absorption in the near infrared, any heat-induced shift in Hb species, such as the formation of methemoglobin (metHb), hemichromes, or hemoglobin products with different electronic structure, produces measurable spectral changes. In particular, the reaction of Hb with heat depends on its oxygenation state. Oxyhemoglobin (oxyHb) at high temperatures undergoes autoxidation and ferrous iron ${\mathrm{Fe}}^{2+}$ becomes ferric ${\mathrm{Fe}}^{3+}$, producing metHb^22^ or hemichromes.^23^^,^^24^ In contrast, the reaction of deoxyhemoglobin (deoxyHb) with heat under hypoxia follows a different path. Without oxygen, the ferrous iron is not autoxidized and instead forms ferrous hemichromes.^25^ This form is a more stable species than the one originating from oxyHb. Upon re-exposure to oxygen, it readily reacts and forms the ferric species, converging to the same hemichromes present in the oxyHb case.^25^

Our previous work^26^ aligned with these findings and revealed distinct spectral features for the treated deoxyHb. We observed these features during the thermal treatment of *ex vivo* tissues in $\mu_{a}$ spectra. *Ex vivo* tissues are deoxygenated after blood supply interruption, and therefore, in the thermal treatments shown in Ref. 26, only deoxyHb was present. We observed two novel absorption peaks at 770 and 830 nm, which were absent in other near-infrared studies on ablated tissue.^27^^–^^31^ A likely explanation is that most of the previous near-infrared spectroscopy work relied on continuous-wave diffuse optics. These techniques are heavily influenced by the surface properties of the tissue, which is in contact with air and therefore oxygen and may disregard deeper optical features. Significantly fewer works employed time-domain methods, which offer a much higher surface rejection.^32^ In fact, to our knowledge, the first work to report these novel peaks is by Lanka et al.,^33^ who utilized time-domain diffuse optical spectroscopy (TD-DOS) over the wide wavelength range of 650 to 1100 nm.

Therefore, we aim to clarify the spectral pathways of Hb under controlled heating and oxygenation conditions. Blood phantoms^34^^–^^36^ offered the most reliable system, as they lack additional compounds that could interfere with heme proteins. Furthermore, oxygenation and deoxygenation could be controlled in *ex vivo* blood, enabling reproducible experiments. In contrast, *ex vivo* organs posed significant limitations: oxygenation required destructive processing, and tissue blending inevitably exposed samples to air, preventing accurate control of their oxygenation state. In this work, we aim to demonstrate that the 770- and 830-nm absorption peaks,^26^^,^^33^ emerging during *ex vivo* thermal treatment, result from the reaction of deoxyHb and do not form in oxygenated environments. As shown here, these bands correspond to the early onset of the more fully developed peaks at 774 and 874 nm that emerge under controlled heating. Based on this hypothesis, we aim to demonstrate that there is an intermediate step in the formation of denatured oxyHb from deoxyHb and that oxygen plays a critical role in the conversion. Moreover, we quantify the extinction spectra for these intermediate denatured states. These insights bridge the gap between *ex vivo* and *in vivo* conditions and open the way for more accurate optical monitoring during thermal therapies.

The remainder of the article is organized as follows. The first section presents the experimental setup, detailing the use of a TD-DOS spectrometer, the sample preparation process, the modeling of reaction dynamics to pinpoint the completion of reactions, and the method for extracting extinction coefficients. The second section presents the results from blood treatment, emphasizing the observed changes in optical properties and their implications for identifying intermediate chromophore states. The final section discusses the implications of the experimental results and the broader relevance of these findings, addresses the limitations of the current experimental setup, and explores how this work advances the understanding of denatured Hb formation and its potential applications in thermal therapies.

## 2 Materials and Methods

### 2.1 Setup and Analysis of TD-DOS

**Fig. 1:**
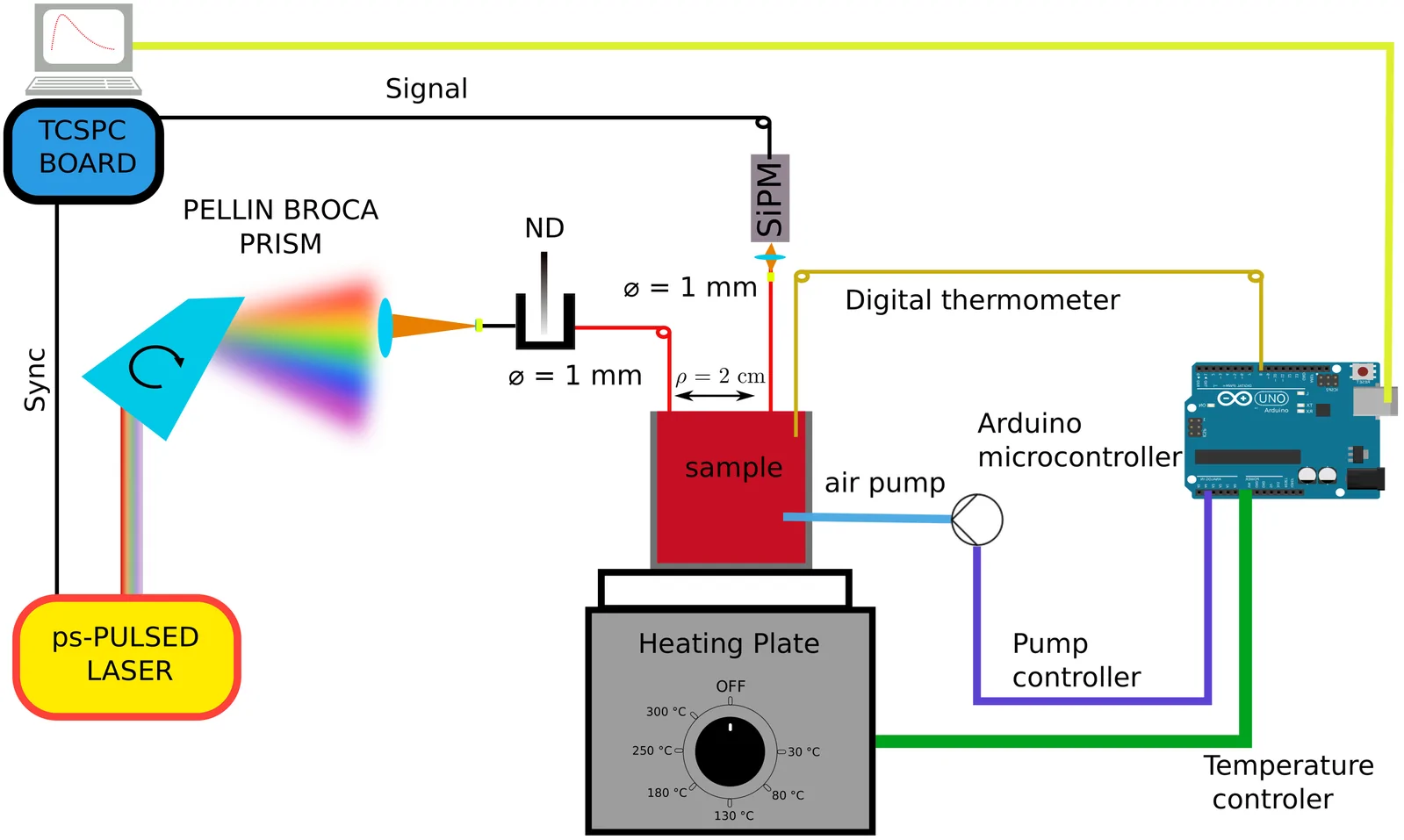
Scheme of the setup employed in the measurements. A picosecond supercontinuum laser (NKT photonics, Fir 20, with a custom fiber) is dispersed by a Pellin Broca prism, which is rotated by a stepper motor, to select a single wavelength. The light is attenuated by a neutral density filter and directed with fiber optics in the sample, where light undergoes diffusion. The backscattered optical signal is then collected by another fiber optic cable at a distance of ρ=2  cm and sent to an SiPM. The distribution of time of flight is reconstructed with a time-correlated single-photon counting board (SPC-130, Becker & Hickl, Berlin, Germany). The sample is treated with a heating plate with a stirrer. The temperature of the heating plate is controlled by an Arduino microcontroller, which is also tasked with recording the temperature of the sample with a digital thermometer. Another Arduino handles a system of peristaltic pumps used to flush air inside the sample.

Figure 1 illustrates the experimental setup. The first component is a time-domain broadband spectrometer^33^^,^^37^^,^^38^ designed for measurement of optical properties ($\mu_{a}$ and $\mu_{s}^{\prime }$), and the second component regulates the temperature of a heating plate to perform thermal treatment of the sample and records the temperature.

The TD-DOS system employs a supercontinuum laser (Fir 20 with a custom fiber, NKT Photonics, Birkerød, Denmark) that generates light pulses of tens of picoseconds at a repetition of 40 MHz in a broad wavelength range (500 to 1750 nm). The light is dispersed by a Pellin Broca prism, which sends the light onto a lens, focusing it onto a 62.5  μmØ core-graded index fiber (NA=0.2). Thanks to the small core dimension, a wavelength bandwidth of around 5 nm is selected, small enough to resolve the absorption lines of common chromophores, and sent to the sample. To change the wavelength, the prism is rotated with a stepper motor controlled by software. To calibrate the motor position, which encodes the wavelength position, we used a spectrometer (USB2000, Ocean Optics, Orlando, Florida, United States). The spectral calibration was performed following the methods outlined in Sec. 2.3. A neutral density filter attenuates the light to ensure single-photon statistics in detection. The light is then coupled into a 1-mm step index silica fiber (NA=0.39) and sent to the sample. An identical fiber collects the diffused light at a distance of 2 cm from the injection fiber and directs it onto a silicon photomultiplier (SiPM). A time-correlated single-photon counting board (SPC-130, Becker & Hickl) processes the analog output of the detector to reconstruct the histogram of the photon distribution of time of flights (DTOF). The measurements were taken from 680 to 1110 nm in 10-nm increments. Each DTOF was integrated for 1 s, and the spectra were acquired every 100 s. The system was validated using the MEDPHOT protocol^39^ and for extreme absorption with ink dilution.^26^

The temperature of the heating plate was controlled using an Arduino-based system, which also monitored and recorded the sample temperature through a digital thermometer (DS18B20) immersed in the sample during the thermal treatment process.

To obtain optical properties ($\mu_{a}$ and $\mu_{s}^{\prime }$) from the collected DTOFs, we used a solution of the radiative transport equation and convolved it with the temporal impulse response function. The inverse fitting procedure was based on a Levenberg–Marquardt least square minimization algorithm using an empirical Mie law derivation to apply a spectral constraint to the scattering spectrum^40^ in the form of $\mu_{s}^{\prime }=A(\lambda /\lambda_{0}{)}^{-b}$ where $\lambda_{0}=650\;\mathrm{nm}$.

The model employed in the fitting procedure consisted of a library of numerically simulated DTOFs with null absorption and variable reduced scattering coefficient^41^ obtained by running a Monte Carlo code for radiative transport of light in a diffusive medium,^42^ along with in-house-built software routines for binning of photons and construction of a lookup table of DTOFs (MC library). The MC library comprised 39 white ($\mu_{a}=0$) DTOFs with $\mu_{s}^{\prime }$ values increasing according to a geometric progression of ratio 1.09 and initial value $1\;{\mathrm{cm}}^{-1}$, chosen to cover a wide range of scattering values (from 1 to $26.4\;{\mathrm{cm}}^{-1}$) within a reasonable simulation time, while avoiding oversampling in the upper range of scattering values. The domain geometry adopted for each curve in the MC library was a voxelized and homogeneous cylinder of height h=5  cm and diameter D≥4ρ, with ρ=2  cm being the source-detector distance, to disregard boundary effects that are negligible in the experimental conditions. Bulk refractive index was n=1.33 to account for the water-based nature of the samples, and the anisotropy factor is g=0.80. The target output counts for each simulated curve were set to $10^{7}$ photons.

### 2.2 Sample Preparation and Experimental Protocol

We used fresh blood rather than commercially available powdered Hb to ensure the Hb retained its original chemistry, free from the effects of chemical processing or high-temperature exposure, preserving its native properties for the experiments. We obtained whole bovine blood from a local slaughterhouse and stored it at 4°C until use. We did not add anticoagulants to avoid chemical interference with the absorption spectra. The blood was allowed to coagulate naturally, and we discarded the uncoagulated portion. To extract Hb, the coagulated mass was lysed with distilled water.

To prepare the phantoms, we added different masses of blood to a solution containing 10 g of Intralipid 20% and 200 g of distilled water. We selected Intralipid as the scattering agent to be consistent with standard literature on liquid^43^ and blood phantom studies.^34^^–^^36^^,^^44^

To oxygenate the phantoms, we bubbled ambient air through the solution, whereas to deoxygenate them, we added dried baker’s yeast^45^ and amides together with glucono-δ-lactone and sodium bicarbonate. The yeast metabolized the amides and consumed dissolved oxygen, and the reaction of glucono-δ-lactone with water of the sample produced gluconic acid, which subsequently reacts with sodium bicarbonate to form sodium gluconate, water, and carbon dioxide, creating an environment favorable for further oxygen depletion. This reaction can be summarized as

$$C_{6}H_{12}O_{7}+{\mathrm{NaHCO}}_{3}\to C_{6}H_{11}O_{7}\mathrm{Na}+H_{2}O+{\mathrm{CO}}_{2}.$$ (1)

The ${\mathrm{CO}}_{2}$-rich environment further promoted oxygen depletion, and the sodium gluconate reduced gas solubility in solution. The preparation temperature was maintained at 30°C to decrease oxygen solubility and enhance deoxygenation efficiency.

For oxyHb measurements, samples were maintained at ambient temperature under continuous aeration for 30 min. Complete oxygenation or deoxygenation was confirmed by comparing measured spectra against published reference spectra of oxy- and deoxyHb.^46^

After spectral confirmation (≈30  min), we set the heating plate temperature to 250°C to start the treatment. Samples were kept at this temperature for 60 min then allowed to cool for 30 min. The temperature of the solution was recorded for each acquired curve (approximately every 1 s), alongside a timestamp.

The container had a diameter of 10 cm, and the solution reached a height of around 5 cm. The probe was immersed at a depth of 1 cm. Given the high absorption of the sample and scattering above $5\;{\mathrm{cm}}^{-1}$, we assume minimal boundary effects in light propagation.

We prepared multiple blood concentrations to enable the extraction of the Hb extinction coefficient: 10 samples for the deoxygenated measurements and 9 for the oxygenated measurements, using processed blood mass fraction ranging from 0.025 to 0.150. The exact recipe for each phantom is included in the openly available dataset.^47^ The total hemoglobin was obtained through spectral fitting as outlined in Sec. 2.3.

### 2.3 Extinction Coefficient Extraction

To determine the molar extinction coefficient, we leveraged the linearity of the Beer–Lambert law, which states

$$\mu_{a}(\lambda )=\underset{i}{\sum }c_{i}\varepsilon_{i}(\lambda ),$$ (2)

where $c_{i}$ represents the concentration of each component, and $\varepsilon_{i}(\lambda )$ denotes the molar extinction coefficient in Naperian units. The following procedure assumes that the sample initially contains only water, oxyHb, or deoxyHb. After the reaction had ended, the sample consisted primarily of water, denatured oxyHb, or denatured deoxyHb.

To ensure accurate calibration of the wavelength axis, it is crucial to account for any potential offsets that might introduce artifacts in the reconstructed extinction coefficients. We first estimated the concentrations of deoxyHb and oxyHb before initiating the treatment by solving the linear system,

$$\mu_{a}(\lambda )=[\begin{array}{cc} \varepsilon_{\mathrm{deoxyHb}}(\lambda ) & \varepsilon_{\mathrm{oxyHb}}(\lambda ) \end{array}][\begin{array}{c} c_{\mathrm{deoxyHb}}\\ c_{\mathrm{oxyHb}} \end{array}],$$ (3)

where $\mu_{a}$ is the absorption coefficient, [ε] denotes the extinction coefficients of deoxyHb and oxyHb, and [c] represents their concentrations. Then, we subtracted the hemoglobin contributions from the spectral data, thus leaving only the spectral signature of water. As water’s spectrum is temperature-dependent,^48^ we compared its spectral centroid with a reference measurement taken at a similar temperature and then applied a shift based on an interpolated curve to align the spectra. We employed a cubic interpolation using *interp1d* from the scipy Python package.

To study the dynamic behavior of the spectra, we analyzed the temporal gradients of the spectral data at wavelengths corresponding to denatured deoxyHb peaks. By calculating these derivatives, we identified a kinetic plateau that indicated the reaction stability, marking significant points where the system reached equilibrium. We consider the denaturation reaction completed at the earliest time t after the start of heating, for which $|d\mu_{a}(\lambda ,t)/dt|<0.044\frac{1}{{\mathrm{cm}}^{-1}s}$, obtained from a preliminary analysis of the evolution of $\mu_{a}$ and the spectral centroid, chosen empirically as the smallest threshold that robustly separates the plateau from the rising phase across all experiments. As Hb is a strong chromophore, and its mass concentration is several orders of magnitude lower than that of water, we assumed the water concentration constant at ≈100%, and from the initial absorption spectra, we subtracted the water contribution at the same temperature, assuming the absorbers to consist solely of oxyHb or deoxyHb and water. There are minor reported temperature dependencies for deoxyHb and oxyHb spectra in the literature,^49^ confined in amplitude to 3% in the temperature range from 20°C to 40°C which we did not take into account.

The spectra were fitted using a linear model: $\mu_{a}(\lambda )=c_{\mathrm{oxy}}\varepsilon_{\mathrm{oxy}}(\lambda )+c_{\mathrm{deoxy}}\varepsilon_{\mathrm{deoxy}}(\lambda )$, where $c_{\mathrm{oxy}}$ and $c_{\mathrm{deoxy}}$ are the concentrations of oxyHb and deoxyHb, respectively, and $\varepsilon_{\mathrm{oxy}}(\lambda )$ and $\varepsilon_{\mathrm{deoxy}}(\lambda )$ their known molar extinction coefficients, respectively. We fitted in the wavelength range of 700 to 900 nm.

To extract the extinction coefficient of denatured Hb, we first subtracted the water contribution at the same temperature from the spectra of treated samples. This ensured that the remaining spectral data exclusively reflected denatured Hb.

For each wavelength $\lambda_{0}$, under the assumption that $\mu_{a}(\lambda_{0})$ is a linear combination of molar extinction coefficients weighted by respective concentrations, we isolated the contribution of denatured Hb. Given that all other components were removed, the extinction coefficient $\varepsilon_{D\text{-}\mathrm{Hb}}(\lambda_{0})$ was calculated as

$$\varepsilon_{D\text{-}\mathrm{Hb}}(\lambda_{0})=\frac{\mu_{a}(\lambda_{0})}{c_{D\text{-}\mathrm{Hb}}},$$ (4)

where $c_{D\text{-}\mathrm{Hb}}$ is the concentration of denatured Hb. Measurements from multiple samples provided the mean extinction coefficient and standard deviation for each wavelength. The mean extinction coefficient represents the estimated value. We calculated the uncertainty at the 95% confidence level using the Student’s *t*-distribution: $U_{95}=t_{0.975,n-1}\frac{s}{\sqrt{n}}$, where (s) is the sample standard deviation, (n) is the number of measurements, and ($t_{0.975,n-1}$) is the two-sided t-value corresponding to a 95% confidence interval with (n-1) degrees of freedom. Multiple repetitions were conducted to ensure robust statistical accuracy and minimize error.

## 3 Results

**Fig. 2:**
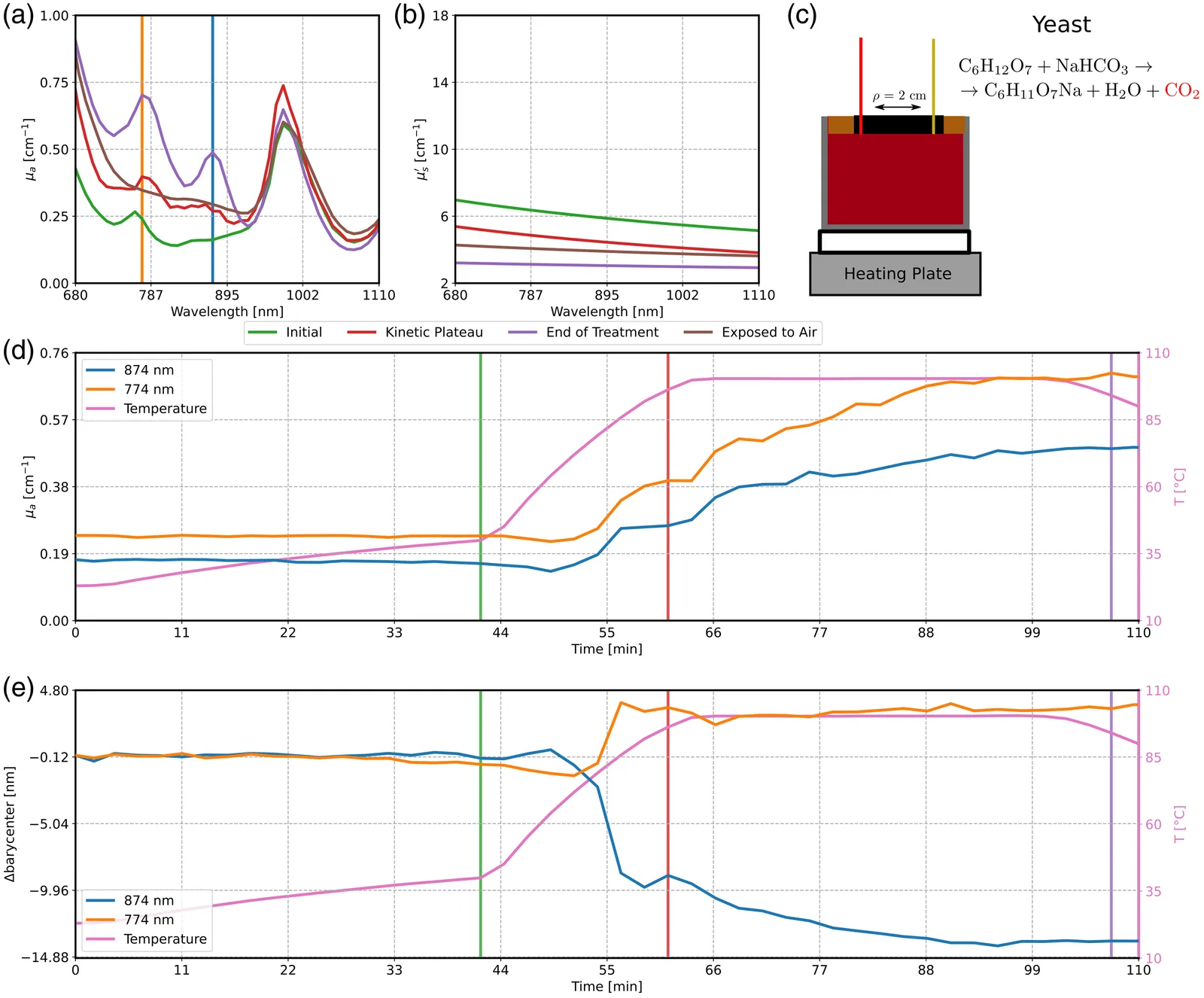
Absorption coefficient $\mu_{a}$ (a) and reduced scattering coefficient $\mu_{s}^{\prime }$ (b) spectra from 680 to 1110 nm of the blood phantom in the deoxygenated case. The measurement “exposed to air” is present only here and not in Fig. 3 because, in that case, all measurements were already performed with full oxygenation. (c) Scheme of the measurement setup alongside the reaction of glucono-δ-lactone employed for sample deoxygenation. (d) Evolution of the absorption spectra at 874 and 774 nm together with the temperature evolution of the sample. The red vertical line is the point of kinetic plateau, when the derivative in time of the absorption coefficient goes below a threshold. Green and purple vertical lines indicate the start and end of the thermal treatment, respectively. (e) Evolution of the spectral centroid of the peaks at 774 and 874 nm with respect to the starting position.

**Fig. 3:**
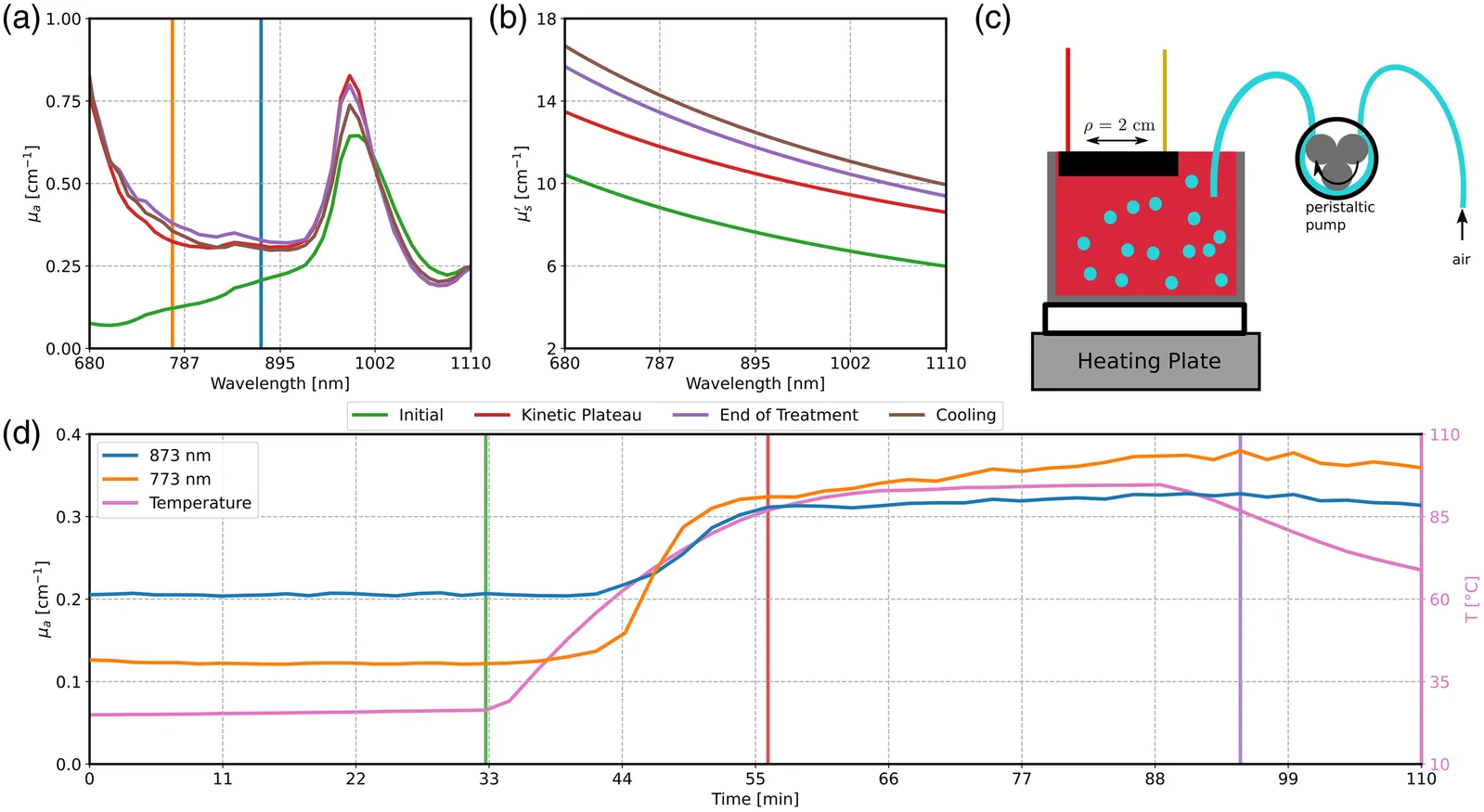
Absorption coefficient $\mu_{a}$ (a) and reduced scattering coefficient $\mu_{s}^{\prime }$ (b) spectra from 680 to 1110 nm during different stages of the thermal treatment (start, kinetic plateau, end, and during cooling) of the blood phantom under full oxygenation. (d) Evolution of the absorption spectra at 874 and 774 nm together with the temperature evolution of the sample. The red vertical line is the point of the kinetic plateau, when the derivative in time of the absorption coefficient goes below a threshold. Green and purple vertical lines indicate the start and end of the thermal treatment, respectively. (c) Scheme of the measurement setup and the oxygenation process of the sample.

Figures 2 and 3 report the $\mu_{a}$ and $\mu_{s}^{\prime }$ spectra of the deoxygenated and oxygenated phantoms during thermal treatment, respectively. Figures 2(d) and 3(d) additionally show the temporal evolution of $\mu_{a}$ at 774 and 874 nm, together with sample temperature. The vertical red marker indicates the time point where the derivative of the absorption coefficient fell below a threshold, defined here as “kinetic plateau” following the approach outlined in Sec. 2.

Sample preparation times differed among conditions: the deoxygenated case required 40 min at 30°C, whereas the oxygenated case required 30 min at 20°C. Both samples reached near boiling in 20 min and were maintained for 60 min. The temperature was maintained below the boiling point to avoid vapor pockets, which may disrupt photon trajectories and affect spectral stability.

The spectra are reported only after 680 nm because, at lower wavelengths, the collected signal was too low because of high absorption during the treatment.

### 3.1 Reduced Scattering Coefficient ($\mu_{s}^{\prime }$)

The reduced scattering spectra ($\mu_{s}^{\prime }$), shown in Figs. 2(b) and 3(b), lack distinct spectral features, as they were obtained assuming the Mie power law. During measurement, it changed with the duration of treatment. In particular, it showed different behaviors in the oxygenated and deoxygenated cases. In the deoxygenated case, in all measurements, the scattering spectrum experienced a significant decrease, whereas in the oxygenated case, it showed a nearly constant value, with a possible increase in the intensity during cooling.

### 3.2 Absorption Coefficient ($\mu_{a}$)

At room temperature (initial state), the spectrum in the deoxygenated case [Fig. 2(a)] corresponds to the overlap of deoxyHb and water absorption, with an increased intensity of the absorption peak at 760 nm, below 700 nm, and a decreased absorption coefficient at 1064 nm as compared with the whole blood case. During the thermal treatment, the absorption spectrum is rapidly altered with the emergence of two peaks at 774 and 874 nm, which are marked at the end of treatment. After cooling and air exposure, these two peaks disappear. The overall absorption reduces; however, it does not return to the initial lineshape but presents a major absorption below 700 nm and no notable peaks after.

In the oxygenated case [Fig. 3(a)], the initial state is the linear combination of oxyHb and water. We report (i) no peak at 760 nm, (ii) a lower absorption below 700 nm compared with the deoxyHb case, and (iii) a higher absorption in the region above 900 nm, including an increase of the valley around 1064 nm. Thermal treatment caused a broad increase in absorption, particularly in the region below 700 nm, but no discrete peaks appeared between 680 and 1100 nm. After cooling, there were no further changes in the features, and the resulting spectrum resembled that of the deoxygenated sample after re-exposure to air.

In both cases, we observed an increase in the absorption at 970 nm with a blueshift of the peak. This process is reversible and temperature-dependent.

To further understand the kinetics of the temporal evolution of thermal treatment, we report in Figs. 2(d) and 3(d) the evolution of $\mu_{a}$ at 774 and 874 nm corresponding to the peaks of absorption in the deoxygenated case.

In the deoxygenated case [Fig. 2(d)], the temporal evolution of the absorption at 774 and 874 nm can be divided into three distinct regimes. First, the signal follows a sigmoidal increase, indicating the progressive transformation of deoxyHb toward a denatured state and approaching saturation. This is followed by a second regime characterized by a continued increase in absorption mainly due to water evaporation, which reduces the sample volume and therefore increases the effective concentration of absorbers. Finally, in the third regime, both peaks continue to increase but with different slopes, with the 874-nm band becoming more prominent relative to the 774 nm band. In contrast, the evolution of the peak spectral centroids [Fig. 2(e)] exhibits only two regimes. The initial sigmoidal phase is associated with minimal spectral shifts, whereas the final phase shows a clear divergence between the two bands. The intermediate regime observed in Fig. 2(d) does not appear in the spectral centroid evolution, as water evaporation affects only the overall intensity through a concentration effect and does not induce spectral shifts. For the 874-nm band, the initial behavior must be interpreted with care: the initial spectrum does not exhibit a well-defined peak but rather a broad shoulder. The initial apparent blue shift observed in Fig. 2(e) therefore reflects the redistribution of spectral weight associated with the progressive emergence and sharpening of a peak. However, this initial phase is also informative through the centroid analysis, as it highlights the spectral changes of the samples.

In contrast, the oxygenated case [Fig. 3(d)] showed a single sigmoidal transition, attributed to oxyHb reaction, followed by a linear increase in intensity that we ascribe to water evaporation.

**Fig. 4:**
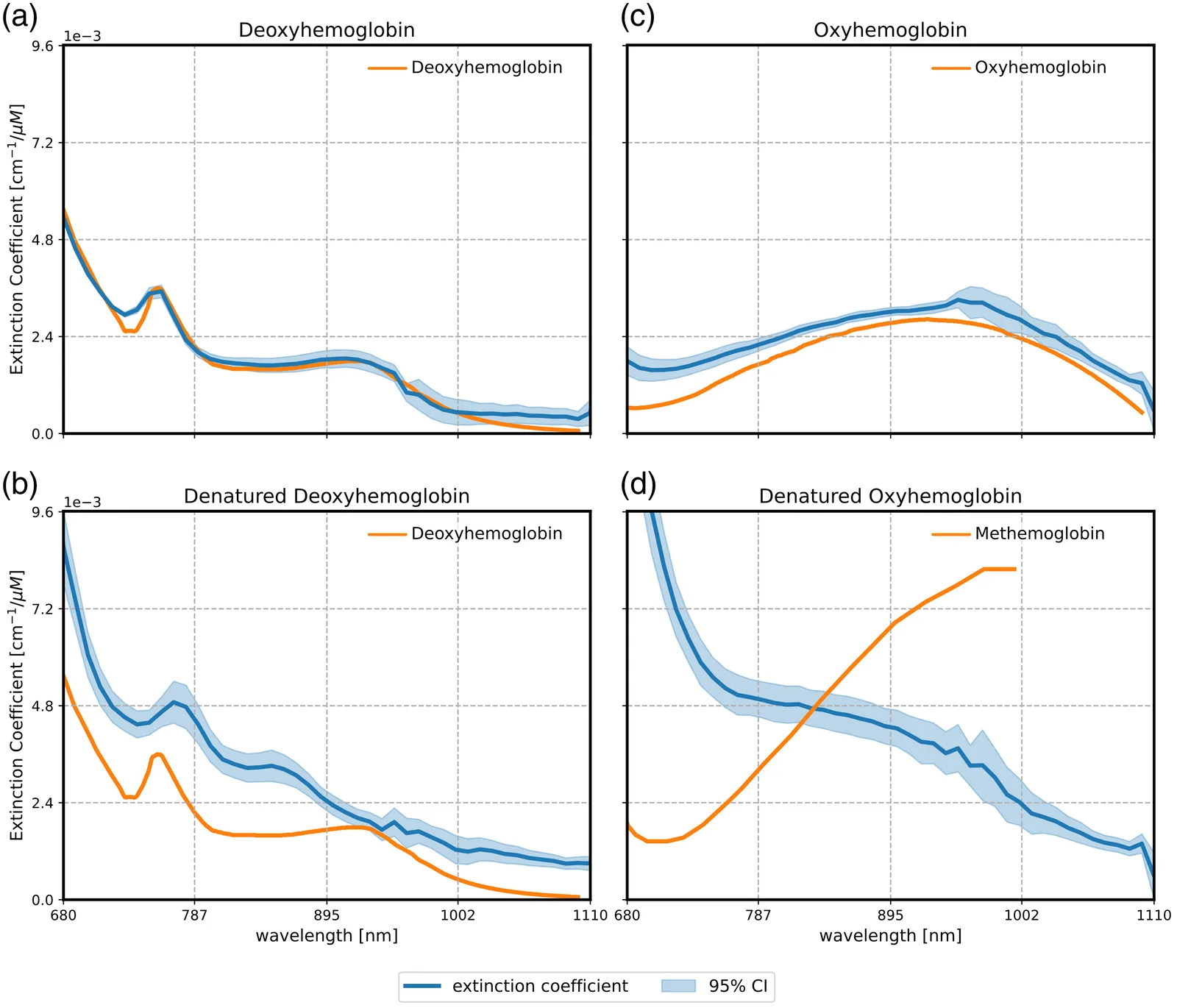
Extracted extinction coefficients (mean±95% confidence interval) for deoxyHb (a), denatured deoxyHb (b), oxyHb (c), and denatured oxyHb (d), compared with the reference spectra of deoxyHb, oxyHb, and metHb. The spectra of metHb were taken from Ref. 50. The extinction coefficient was retrieved by fitting the concentration of hemoglobin from the solution before the treatment and then taking advantage of the linearity of the Beer–Lambert law. We employed 10 repetitions for deoxyHb and 9 repetitions for oxyHb. The 95% confidence intervals were computed using the t-student distribution.

### 3.3 Extinction Coefficient (ε)

Figure 4 shows the extracted extinction coefficient of the four species involved in the process (oxy- and deoxyHb and denatured oxy- and deoxyHb). Native deoxyHb and oxyHb coefficients showed consistent features with literature spectral shapes, although absolute values differed in oxyHb. We successfully retrieved the extinction coefficient of deoxyHb with minimal error. For the oxyHb case, we report identical spectral features compared with the literature case, however, showing different absolute values. The denatured deoxyHb showed clear differences compared with the spectrum of deoxyHb, with a red-shifted peak at 774 nm and a minor peak at 874 nm approximately. This spectrum does not show the major peaks of Fig. 2(a). The denatured oxyHb showed no strong peak above 700 nm and a clear difference from the reference spectra of metHb.^50^

The resulting estimated extinction coefficients showed less than 5% statistical uncertainty across the spectrum, as quantified by the 95% confidence interval. For all cases, we saw a higher variability around 960 nm related to the removal of the water absorption spectra from the measured $\mu_{a}$.

## 4 Discussion

### 4.1 Reduced Scattering Coefficient ($\mu_{s}^{\prime }$)

Although our analysis primarily emphasizes the absorption coefficient ($\mu_{a}$), the reduced scattering coefficient ($\mu_{s}^{\prime }$) plays a crucial role in ensuring the accuracy of the optical properties retrieval. We observed variations in $\mu_{s}^{\prime }$ due to several factors, such as foam from yeast and ${\mathrm{CO}}_{2}$ production and changes in saline solution refractive index in the deoxygenated case. We noted no reduction in the oxygenated case. Placing the source detection distance at 2-cm facilitated sufficient scattering events for accurate absorption data extraction. To enhance accuracy, we employed Monte Carlo simulations and the Mie power law, fitting spectra across 44 measurements for minimal noise. Additional factors contributing to changes in $\mu_{s}^{\prime }$ are the refractive index dependence on temperature and bubbles from oxygenation.

### 4.2 Absorption Coefficient ($\mu_{a}$) and Extinction Coefficient (ε)

The absorption coefficient is a unique fingerprint of a molecule, reflecting its specific electronic and vibrational transitions. Previous studies have shown that the thermal response of Hb strongly depends on its oxygenation state.^22^^,^^24^^,^^30^^,^^51^ Our observations expand on this knowledge by combining chemical pathways with spectral evidence. We found that the thermal treatment of deoxyHb revealed distinct spectral features compared with the treatment of oxyHb, pointing toward a different chemical configuration of Hb.

For oxyHb, heating first decreases oxygen solubility, increasing the proportion of deoxyHb,^30^^,^^51^ confirming that deoxyHb is a transient intermediate between oxyHb and denatured oxyHb. At higher temperatures, oxyHb undergoes autoxidation, in which ferrous iron (${\mathrm{Fe}}^{2+}$) is oxidized to ferric iron (${\mathrm{Fe}}^{3+}$), forming metHb^22^ and hemichromes.^23^^,^^24^ These unstable species can further release hemin, leading to ferric heme aggregation in Heinz bodies.^24^ Importantly, the spectral signatures of denatured oxyHb remain distinct from those of metHb, highlighting that these are separate chemical configurations.

We observe that the denatured deoxyHb follows a path that is spectroscopically distinct from the oxyHb case. We believe that an intermediate form of Hb in the denaturation of deoxyHb different from the one from oxyHb is evidenced by four reproducible features: (a) the emergence of a peak at 874 nm, (b) a red-shift of the 760-nm peak, (c) a significant increase in absorption below 700 nm, and (d) the disappearance of the 774/874-nm peaks upon cooling.

We observe from Fig. 2(d) that the heating of deoxyHb proceeds through a three-step process. This interpretation is further supported by the evolution of the corresponding peak spectral centroids in Fig. 2(e), which reflect the underlying chemical changes: (1) deoxyHb first transitions into denatured deoxyHb, as evidenced in both Figs. 2(d) and 2(e). (2) Evaporation reduces the solvent volume, thereby increasing the relative concentration of denatured deoxyHb. As Hb content is negligible compared with solvent volume (0.1  μM), the solution can be considered almost entirely water; thus, the observed volume reduction is attributed solely to water loss. This effect is visible in Fig. 2(d) as it is not chemically related. (3) Both peaks increase in intensity, with the 874-nm band rising more steeply than the 774-nm band, accompanied by a red shift of the peaks, as shown in Figs. 2(d) and 2(e).

Finally, once the oxygen was again present in the cooled solution of deoxyHb, the spectra were identical to those of treated oxyHb.

The absence of the peaks at 774 and 874 nm in the commonly reported spectra of ablated tissues^28^^–^^30^ and blood,^31^ and its similar, although shifted by tens of nanometers, features to metHb^50^ underscores that the observed spectral changes correspond to a distinct chemical configuration of Hb in the hypoxic case which is different from the one commonly found. Based on these findings, we suggest the presence of a ferrous-denatured Hb, due to its spectral resemblance to deoxyHb and supporting evidence from Ref. 25. According to Ref. 25, without oxygen, the ferrous iron does not undergo autoxidation but instead forms a more stable intermediate, which we identify as ferrous-denatured Hb. Upon exposure to oxygen, in this case caused by the increased solubility of gases in water at lower temperatures, which enhances the availability of oxygen in water and converts it to ferric species, converging to the same hemichromes pathway as in the oxyHb case. In fact, the spectra converge with those of denatured oxyHb, consistent with conversion of the ferrous intermediate into ferric hemichromes.

The different dynamics of the 774 to 874-nm peaks are consistent with the formation of hemichromes and Heinz bodies,^24^ as the disproportionate growth of the 874-nm peak compared with the one at 774 nm cannot be explained by concentration effects alone. Thus, the reported extinction coefficient, we believe, does not consider all the components but only a linear combination of molecules. Importantly, these spectral dynamics also clarify the apparent discrepancy between the 830-nm peak reported in Refs. 26 and 33 and the 874-nm peak observed here. The earlier *ex vivo* studies employed lower temperatures or shorter heating durations, leading to a partial development of the same bands, manifested earlier in their evolution near 830 nm. Under the more controlled and prolonged heating conditions used in the present work, these features progress to their fully developed positions at ∼774 and 874 nm.

Our previous studies^26^^,^^33^^,^^52^ were, to the best of our knowledge, the first to report the two peaks at 770 and 830 nm. Other optical methods, such as hyperspectral imaging or continuous wave diffuse optics, are much more affected by the tissue surface than time-domain methods,^32^ and this could be the reason why the two peaks were not observed previously. In *ex vivo* tissue, the surface is oxygenated due to air exposure during storage, whereas deeper tissue layers are entirely deoxygenated. TD-DOS relies on the photon propagation path within the tissue. The surface layer’s contribution to the signal is weighted proportionally to the photons’ travel path through it, allowing for differentiation between surface and depth information.

The identification of a novel chromophore spectrum has several applications in the thermal therapy field: (i) This finding further validates existing results in the study of thermal denaturation of Hb.^23^^–^^25^^,^^53^ (ii) To distinguish the *in vivo* from the *ex vivo* case: although both may show initially similar spectra, oxygen is readily available *in vivo*, whereas in *ex vivo* cases, the oxygen is completely depleted as cells consume it before dying. This oxygen loss limits the relevance of *ex vivo* studies, as it can significantly alter Hb behavior. Consequently, to accurately model this phenomenon in phantoms, during preparation, a careful control of oxygen presence is essential to ensure physiologically accurate results. (iii) Accurate laser ablation modeling requires precise tissue absorption spectra to model light distribution and heat delivery, and explicitly accounting for the *ex vivo* scenario, in test measurements, and *in vivo* scenario, in real surgical ablation. (iv) The treatment may induce local hypoxia by blocking blood flow, which should be accounted for in the modeling. At the same time, hypoxia could be induced, similarly to transcatheter arterial chemoembolization (TACE),^54^ for laser ablation and leveraged therapeutically by exploiting the absorption changes during therapy for precise intervention and by targeting the new peaks or valleys.

More broadly, these findings highlight that optical signals acquired during thermal treatment reflect not only temperature variations but also underlying biochemical transformations. This is especially relevant for the detection of the treatment endpoint, where the clinical objective is to identify when tissue has undergone irreversible alteration rather than simply elevated temperature. By linking spectral features to specific biochemical states, optical monitoring approaches may improve the identification of tissue experiencing irreversible damage, transition zones, and viable regions, thereby contributing to a more accurate definition of treatment margins and reducing the risk of under- or over-treatment. In this perspective, the observed bands at 774 and 874 nm may indicate regions that have undergone complete thermal denaturation. However, the primary clinical challenge still lies in identifying the surrounding transition zone, where tissue may still be viable or undergo delayed processes such as apoptosis. Accurately distinguishing this intermediate region from both necrotic and healthy tissue is critical for effective treatment planning and outcome prediction. Finally, these spectral signatures may also be exploited within multimodal monitoring frameworks combining diffuse optics with complementary techniques such as ultrasound, MR thermometry, or Raman spectroscopy, where they could provide biochemical specificity that is not accessible through temperature measurements alone.

### 4.3 Limitations and Future Directions

Several uncontrolled variables could, in principle, influence the interpretation of the 774- to 874-nm peaks. The experimental limitations that we identified are as follows: (i) We lacked control of pH, dissolved $O_{2}$, and ${\mathrm{CO}}_{2}$, and this limited the quantitative precision of our measurements. (ii) Achieving the denatured deoxyHb state required a carefully controlled deoxygenation process. Simply ensuring the presence of deoxyHb and the absence of oxyHb was insufficient. To mitigate this, we excluded datasets where the two characteristic peaks did not appear. (iii) Water evaporation changed the chromophore concentrations over time, complicating extinction coefficient estimation. (iv) The open container permitted oxygen interaction, potentially shifting spectra toward oxy-derivatives. Residual oxygen, even at trace levels, could bind to denatured deoxyHb, given the high affinity of ferric heme states. (v) Chemical interactions between solution components and Hb cannot be fully excluded. However, yeast itself cannot be directly responsible, as it is inactivated at high temperatures and its mitochondrial cytochromes are present in negligible amounts. Moreover, a control measurement containing only Intralipid, water, yeast, and gluconate salt showed spectra consistent with water alone thus excluding any effects associated with the deoxygenation agents. Gluconate salt produced during the reaction is negatively charged and could form weak bonds with the iron in the heme group, but we believe it is insufficient to account for the reported spectral changes. (vi) Wavelength fine-tuning might add an uncertainty, which we mitigated by a global water–band alignment.

The limitations that we identified in the data analysis are as follows: (i) Calibration uncertainties near water absorption bands further increased the error. (ii) Our extinction coefficient estimates depend on an initial deoxyHb and oxyHb concentration; errors in the initial oxy/deoxy fit propagate to ε, particularly oxyHb’s extinction coefficient. (iii) We are not considering the presence of different molecules in the solution. (iv) Using a constraint on $\mu_{s}^{\prime }$ may cause a cross-talk between $\mu_{s}^{\prime }$ and $\mu_{a}$.

Taken together, these factors may affect the quantitative precision of extinction coefficients and the specificity of peak assignment. Nevertheless, the spectral features observed in the *ex vivo* tissue case,^26^^,^^33^ identical to those reported in the presented solution, strongly support the existence of a distinct Hb intermediate in the transition from deoxyHb to the denatured oxyHb.

Despite these controls, the assignment of the 774- to 874-nm bands to a ferrous intermediate remains putative. Confirming this hypothesis will require further analysis in controlled environments. We attribute the observed peaks to electronic transitions rather than vibrational modes, consistent with the mechanisms described for deoxyHb and oxyHb in Ref. 55 and the high cross-section. However, definitive evidence requires the electronic absorption spectra in the visible and ultraviolet region to identify the electronic transitions present. In addition, the spin state of the molecules through electron paramagnetic resonance^23^ should be evaluated. Vibrational spectroscopy, in particular, Raman spectroscopy,^56^ could further strengthen this assignment by probing the heme bond vibration and monitor structural changes during the deoxyHb denaturation. Diffuse Raman spectroscopy^57^^,^^58^ opens this possibility to measure on real tissue without external oxygen interaction and to identify chemical specimens at various depths.

## 5 Conclusion

In this work, we measured the absorption spectra ($\mu_{a}$) of deoxyHb and oxyHb during a thermal treatment using an Intralipid blood phantom, showing distinct spectral evolution with temperature. We observed the appearance of two peaks in deoxyHb at 774 and 874 nm, which disappear upon air exposure, leaving the spectra identical to those of thermally treated oxyHb. These findings provide evidence that the peaks originate from Hb and are in line with previous reports that deoxyHb denatures into hemichromes, consistent with a ferrous-denatured intermediate and distinct from the one originating from oxyHb. Beyond confirming this mechanism, our study provides the spectra of this chromophore, fully available as an open dataset.^47^ This has important implications for thermal therapy: it highlights the crucial differences between *ex vivo* and *in vivo* oxygen availability. Furthermore, accurate knowledge of these absorption features is essential for realistic light–tissue interaction models in laser ablation.

## Data Availability

According to the open data policies of the Physics Department of Politecnico di Milano, all data underlying the results presented in this paper are publicly available on Zenodo^47^ under CC-BY license, DOI: 10.5281/zenodo.17739078.
